# An antigen panel to assess the regional relevance of foot and mouth disease vaccines

**DOI:** 10.1038/s41541-025-01128-7

**Published:** 2025-05-26

**Authors:** David J. Paton, Ginette Wilsden, Clare FJ Browning, Efrem A. Foglia, Antonello Di Nardo, Nick J. Knowles, Jemma Wadsworth, Simon Gubbins, Ethel Chitsungo, Cisse Rahamatou Moustapha Boukary, Gelagay Ayelet, Charles S. Bodjo, Nick Nwankpa, Emiliana Brocchi, Santina Grazioli, Anna Ludi, Donald P. King

**Affiliations:** 1 The Pirbright Institute, Pirbright, Woking, Surrey, United Kingdom; 2 Istituto Zooprofilattico Sperimentale della Lombardia e dell’Emilia Romagna, Brescia, Italy; 3 The African Union Pan African Veterinary Vaccine Centre (AU-PANVAC), Addis Ababa, Ethiopia

**Keywords:** Immunology, Microbiology, Diseases

## Abstract

Despite widespread use of inactivated vaccines to control foot-and-mouth disease (FMD), there is no systematic approach to demonstrate the regional relevance of these products against the specific serotypes and strains that circulate in endemic countries in Africa and Asia. Failure to adopt independent testing of FMD vaccines has contributed to poor trust in their quality and a lack of investment in vaccination programmes. Therefore, a reference antigen panel representing four serotypes, tailored for East Africa, has been established and used to measure FMDV-specific antibody responses in cattle after administration of FMD vaccines commercially available in the region. This revealed inconsistencies and gaps in cross-neutralisation responses that are evident for some vaccines even after giving booster doses. It is concluded that the East Africa reference antigen panel can be used to evaluate FMD vaccine potency and drive up vaccine quality. Further panels could be developed and deployed for other endemic regions.

## Introduction

Foot-and-mouth disease (FMD) is an economically important vesicular disease that primarily affects domestic and wild cloven-hoofed animals. The causative virus (FMDV) is a member of the family *Picornaviridae* in the genus *Aphthovirus* and exists as multiple serotypes and strains. The contagious nature of the disease, its ability to spread by different routes and hosts, and its evolving antigenic diversity make control difficult^1^. Although eradicated in some parts of the world, FMD has a global distribution, and the disparities in regional status with respect to FMD occurrence have a profound impact on global trade in livestock and their products. Commercial FMD vaccines are produced from inactivated virus and elicit a serotype-specific and relatively short-lived protection that may also be incomplete against antigenically divergent strains of the same serotype^2^. Nevertheless, vaccination remains an important control measure, and more than two billion FMD vaccine doses are given annually, either prophylactically or in response to disease outbreaks^3^.

Producing consistently high-quality FMD vaccines to cover the diversity of serotypes and antigenic variants in Africa is challenging and requires viruses to be grown to high titre in expensive biosecure facilities^3^. The active antigen, the FMDV capsid (146S), has low stability, readily dissociating into smaller sub-components (12S) with greatly diminished immunogenicity^4,5^. Stringent quality controls are therefore needed, including tests of vaccine efficacy. As there is a correlation between the protection provided by FMD vaccines and the amount of antibody they elicit, serology is widely used as a predictor of efficacy^3,5–9^. When a new vaccine strain is registered, evidence of efficacy is usually provided by a potency test involving vaccination and subsequent challenge of the target livestock species to measure protection directly, using the same strain of virus in the challenge as included in the vaccine, i.e., homologous challenge. If a correlation can be shown between antibody titres in sera collected at the point-of-challenge and protection, then a threshold serological titre can be chosen for acceptance of future vaccine batches when tested for immunogenicity without challenge^9^. Serological matching tests are also used to determine if a vaccine is likely to provide protection against different strains of the same serotype, usually by reacting monospecific vaccine antiserum with the vaccine (homologous) and field (heterologous) viruses and comparing the titres to derive a one-way relationship value or r_1_ value^9,10^. However, protection will depend upon a combination of antigenic match, vaccine potency, and dose regime, the combined effects of which can be measured directly in a heterologous potency or cross-protection test by field virus challenge of vaccinated animals^7,8^. As such, studies cannot be performed routinely; heterologous serology (“in vitro cross-protection”) may be used instead, with thresholds of acceptance derived from studies of the correlations shown between cross-protection and in vitro serological cross-neutralisation^8^. Africa harbours the widest range of FMDV serotypes and strains, but there are no agreed FMD vaccine strains for use, and there is a lack of independent quality control of the FMD vaccines that are available^11^. When combined with problems and gaps in vaccination programmes, this leads to a lack of trust in FMD vaccines and vaccination. In this study, an FMDV reference antigen panel that is tailored for East Africa has been developed and used to evaluate the potency of FMD vaccines intended for sale in the region. The panel represents the current regional antigenic diversity to enable an assessment of cross-reactive antibody responses of the FMD vaccines against field viruses. This panel of 16 viruses was used in virus neutralisation tests to measure post-vaccination responses in cattle vaccinated with 13 batches of commercial multivalent FMD vaccines, providing data to demonstrate the regional relevance of these vaccines for use in East Africa.

## Results

### Antigen panel selection

**Fig. 1:**
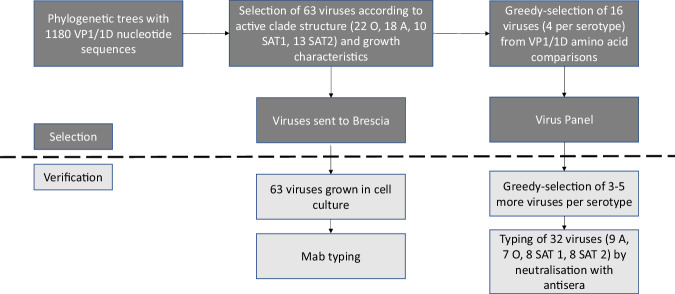
Flow chart summarising the procedure for the selection of the East Africa reference antigen panel of viruses. Use of phylogeny and antigenic typing with monoclonal and polyclonal antibodies to select viruses representative of the diversity of FMDVs found in East Africa.

**Fig. 2:**
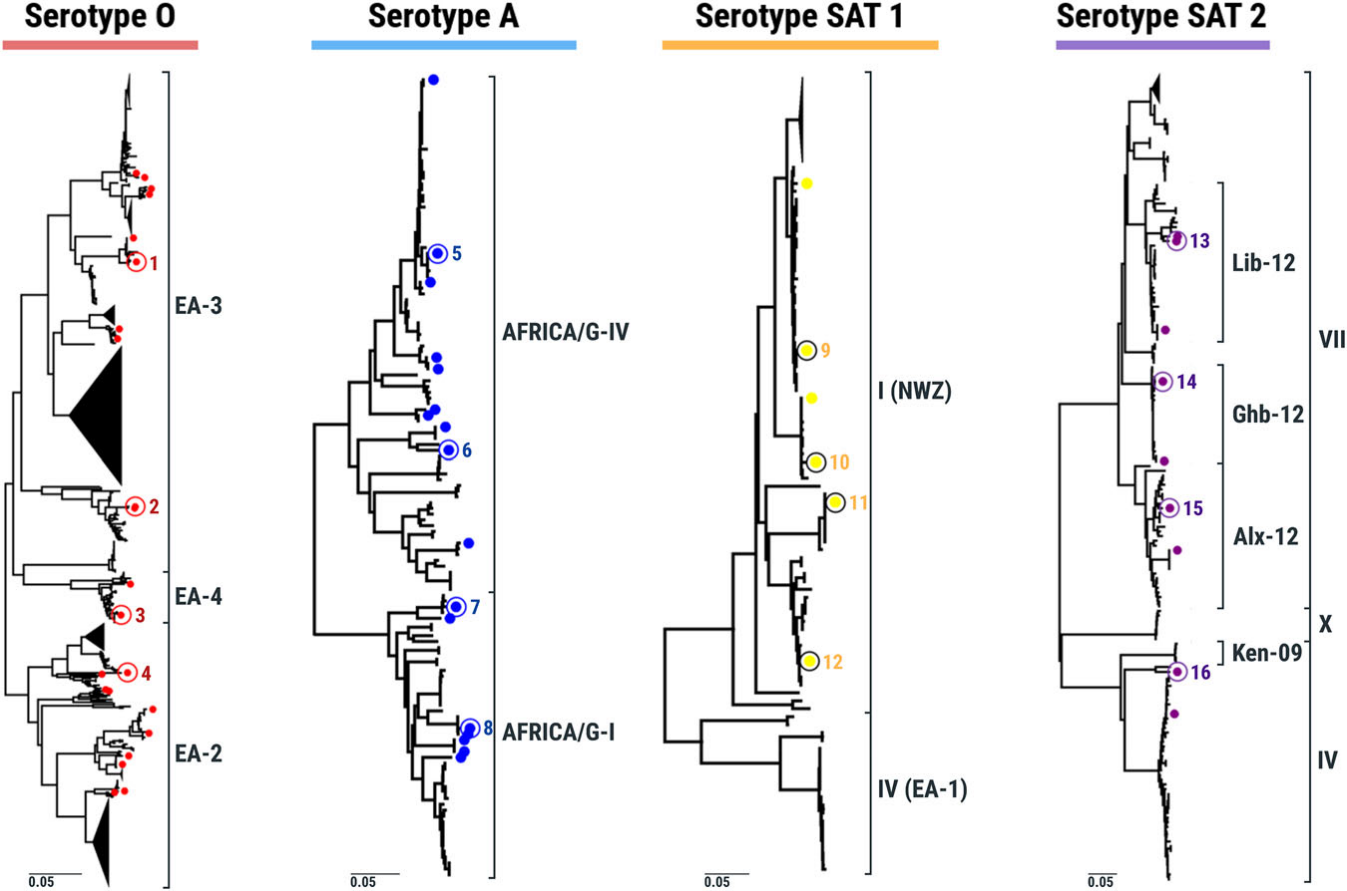
Phylogenetic representation (based on VP1 coding sequences) of FMDV lineages circulating in East Africa. Candidate reference antigens are indicated by the coloured dots, from which the 16 reference antigens (highlighted by circles and numbered as in Table 1) in the East Africa panel were selected. The reconstructed trees also include sequences for FMD viruses isolated from other African regions, which were excluded from the selection process.

**Fig. 3:**
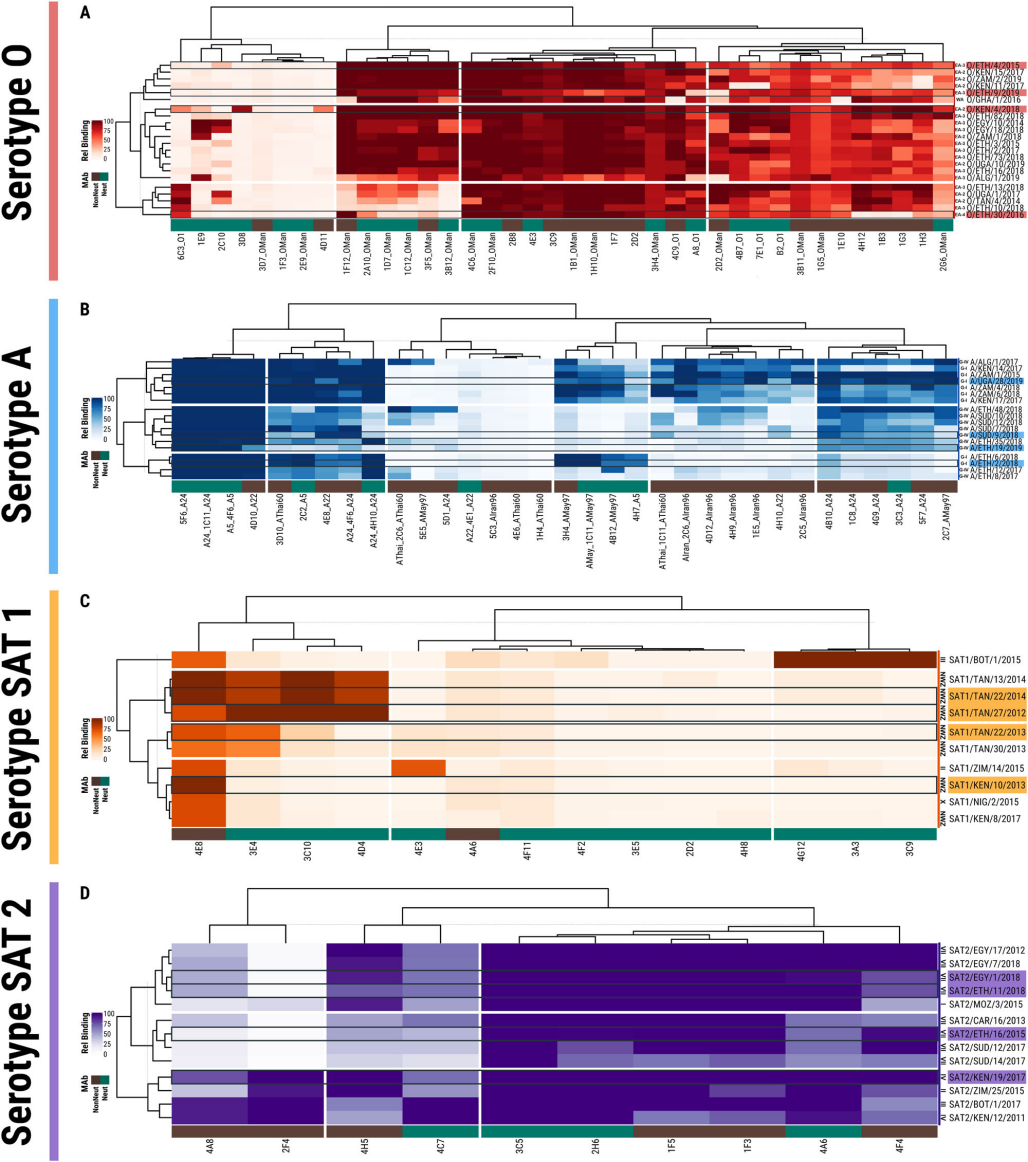
Antigenic relationships revealed by Mab typing. The order of both row and column items is defined by modular leaf ordering methods and based on the smallest average distance of the estimated subtrees. The percentage of reactivity is normalised and expressed on a scale from 0 to 100. The panel viruses are highlighted with delineated reactivity pattern borders. For each serotype the four highlighted viruses are those selected for the final panel. **A** Results for serotype O viruses, typed with Mabs raised against different O strains (7E1 and 4B7 were raised against O/Italy/93, whilst the others were raised against O/Switzerland/1965, O/UKG/2001 or O Manisa as indicated in the Mab name). **B** Results for serotype A viruses, typed with Mabs raised against different A strains (as indicated in the Mab name). **C** Results for serotype SAT 1 viruses, typed with Mabs raised against SAT 1/BOT 1/68. **D** Results for serotype SAT 2 viruses, typed with Mabs raised against SAT 2/ZIM/5/81.

**Fig. 4:**
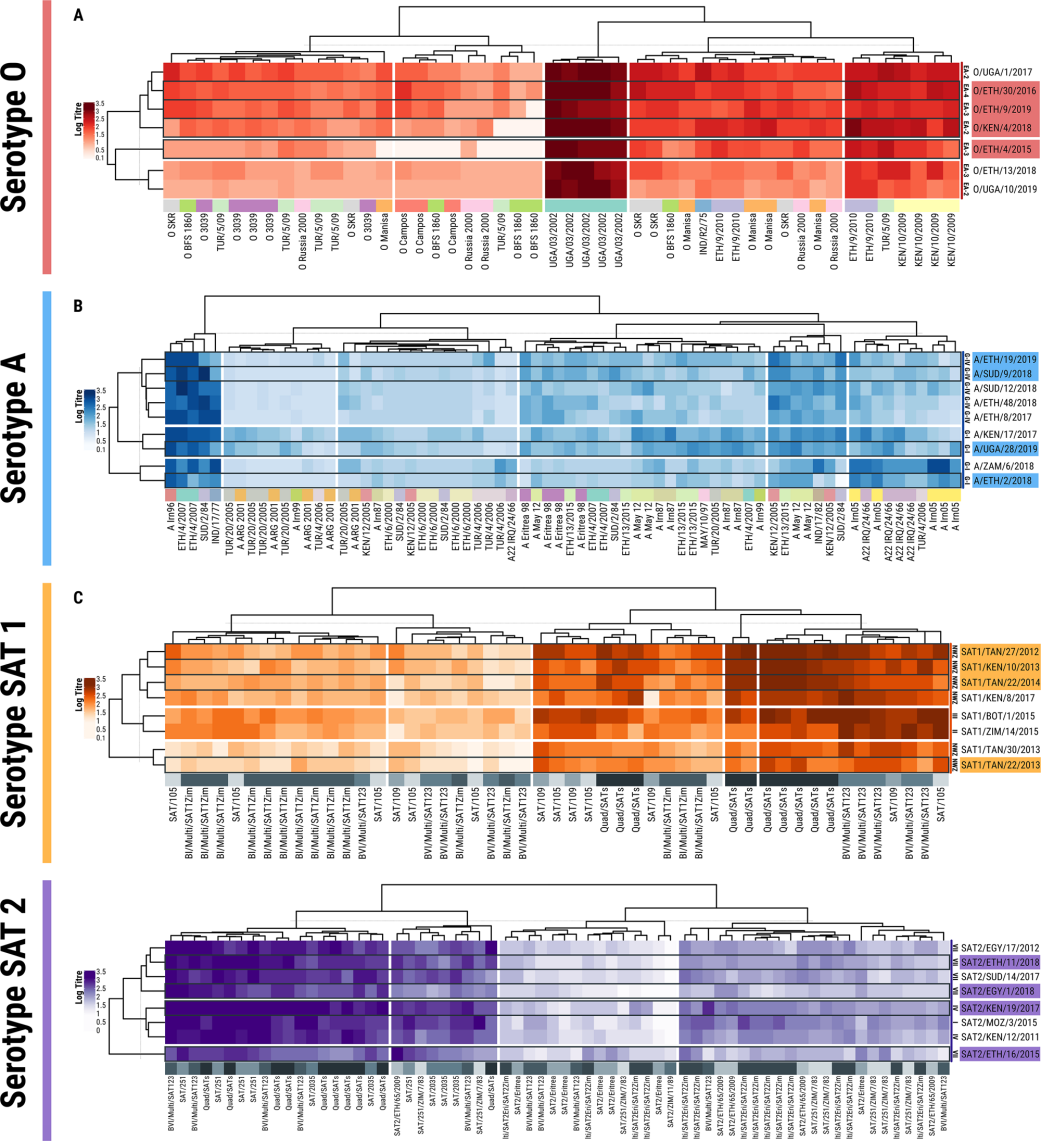
Antigenic relationships revealed by neutralisation tests. The antibody titres are expressed in log_10_, with the test viruses and their associated topotypes listed on the right-hand vertical axis. The different sera are on the x axis and named and coloured according to the vaccine strain against which the antiserum was raised. Duplicated sera are from different cattle. The order of both row and column items is defined by modular leaf ordering methods and based on the smallest average distance of the estimated subtrees. The panel viruses are highlighted with delineated reactivity pattern borders. **A** serotype O; **B** serotype A; **C** serotype SAT 1; **D** serotype SAT 2.

The procedure to select representative FMDV antigens for the reference antigen panel using genetic and antigenic analyses is summarised in Fig. 1, and the final panel composition (four strains for each of the four FMDV serotypes) is shown in Table 1. Figure 2 shows the derived phylogenies for each serotype (1180 viruses in total) from which representatives of contemporary clades were selected. Heatmaps representing the antigenic similarity of the selected viruses revealed by typing with monoclonal antibodies (Mabs, 63 viruses) are depicted in Fig. 3, with Fig. 4 showing the antigenic relationships revealed by virus neutralisation tests (VNT, 32 viruses) (unanalysed results data deposited in Dryad (see Data Availability)). For each serotype, the isolates could be subdivided into several antigenic clusters, with variable congruence to their genetic relationships. Two genetic topotypes predominate for serotype O (East Africa 2 and East Africa 3), but both can be found in all the antigenic clusters defined by Mabs. For serotype A, however, the viruses from topotypes G-I and G-IV are mainly in separate antigenic clusters. Only topotype I of Southern African Territories (SAT) 1 viruses have been isolated since 2007 in East Africa, and so are the only topotypes included in the panel. The two SAT 1 topotype II and III viruses nevertheless appear antigenically distinct. Topotype-related antigenic clustering is also seen for the examined SAT 2 viruses.

**Table 1:** FMDV isolates in the East Africa reference antigen panel

Antigenic reference virus no.	Virus name	Virus lineage	Sequence accession number
1	O/ETH/9/2019	O/EA-3	MT602083
2	O/ETH/4/2015	O/EA-3	MT602081
3	O/ETH/30/2016	O/EA-4	MT602082
4	O/KEN/4/2018	O/EA-2	MT602084
5	A/ETH/19/2019	A/AFRICA/G-IV	MT602078
6	A/SUD/9/2018	A/AFRICA/G-IV	MT602079
7	A/UGA/28/2019	A/AFRICA/G-I	MT602080
8	A/ETH/2/2018	A/AFRICA/G-I	MT602077
9	SAT 1/TAN/22/2014	SAT 1/I	MT602088
10	SAT 1/TAN/22/2013	SAT 1/I	MT602087
11	SAT 1/KEN/10/2013	SAT 1/I	MT602085
12	SAT 1/TAN/27/2012	SAT 1/I	MT602086
13	SAT 2/ETH/11/2018	SAT 2/VII/Lib12	MT602091
14	SAT 2/EGY/1/2018	SAT 2/VII/Ghb12	MT602089
15	SAT 2/ETH/16/2015	SAT 2/VII/Alx12	MT602090
16	SAT 2/KEN/19/2017	SAT 2/IV	MT602092

Mab recognition within each serotype was highly variable, ranging from identical patterns of binding for the antibodies to examples where there was only partial or very poor conservation of the epitopes. For example, many of the SAT 1/BOT/1/68 Mabs recognised only the parental virus (Fig. 3c), whilst for the other serotypes, several Mabs reacted with all the viruses tested. All the panel viruses had unique reactivity profiles and represented all the antigenic clusters containing viruses from East Africa (three principal clusters for each serotype). Mab typing identified a fourth cluster for SAT 1, which comprised a singleton SAT 1 virus from southern Africa (Fig. 3c).

Likewise, neutralisation data also showed that the panel viruses represented the principal clusters for all East Africa viruses across the four serotypes (2, 3, 2, 3 clusters for serotypes O, A, SAT 1 and SAT 2, respectively), although the specific isolate composition and topology of the groups defined by these two approaches was not always identical. In common with the Mab typing data, neutralisation data also suggested that some SAT 1 viruses from southern Africa are antigenically distinct (Fig. 4c).

### Immunogenicity trials on vaccines for use in East Africa

#### Neutralising antibody responses

To rule out prior undisclosed infection with FMDV, the cattle sourced in FMD-endemic settings were confirmed seronegative by ELISA for antibodies to FMDV non-structural proteins (NSP) before vaccination. However, some pre-vaccination sera from such cattle (batches 3, 4, and 5) showed VNT titres (≥1 in 32) above the background levels found in cattle from FMD-free regions and, therefore, the post-vaccination responses of these cattle were excluded from our analyses. This reduced the number of cattle used for analysis of batches 3, 4, and 5 from 10 to five, nine, and five, respectively. Additionally, for batch 4, one cattle serum was missing at the 21-dpv collection point. Results for batch 10 indicated significant pre-existing immunity to serotypes O and A, most likely due to prior vaccination, considering the NSP antibody negative findings. This may have boosted the immunogenicity measured for this batch, a problem that might also have affected the batch 9 trial undertaken by the same manufacturer in the same endemic setting, and for which pre-vaccination sera were not available for testing. Details of the analysis of the pre-vaccination serostatus are set out in Supplementary Note 1 and Supplementary Table 1.

**Fig. 5:**
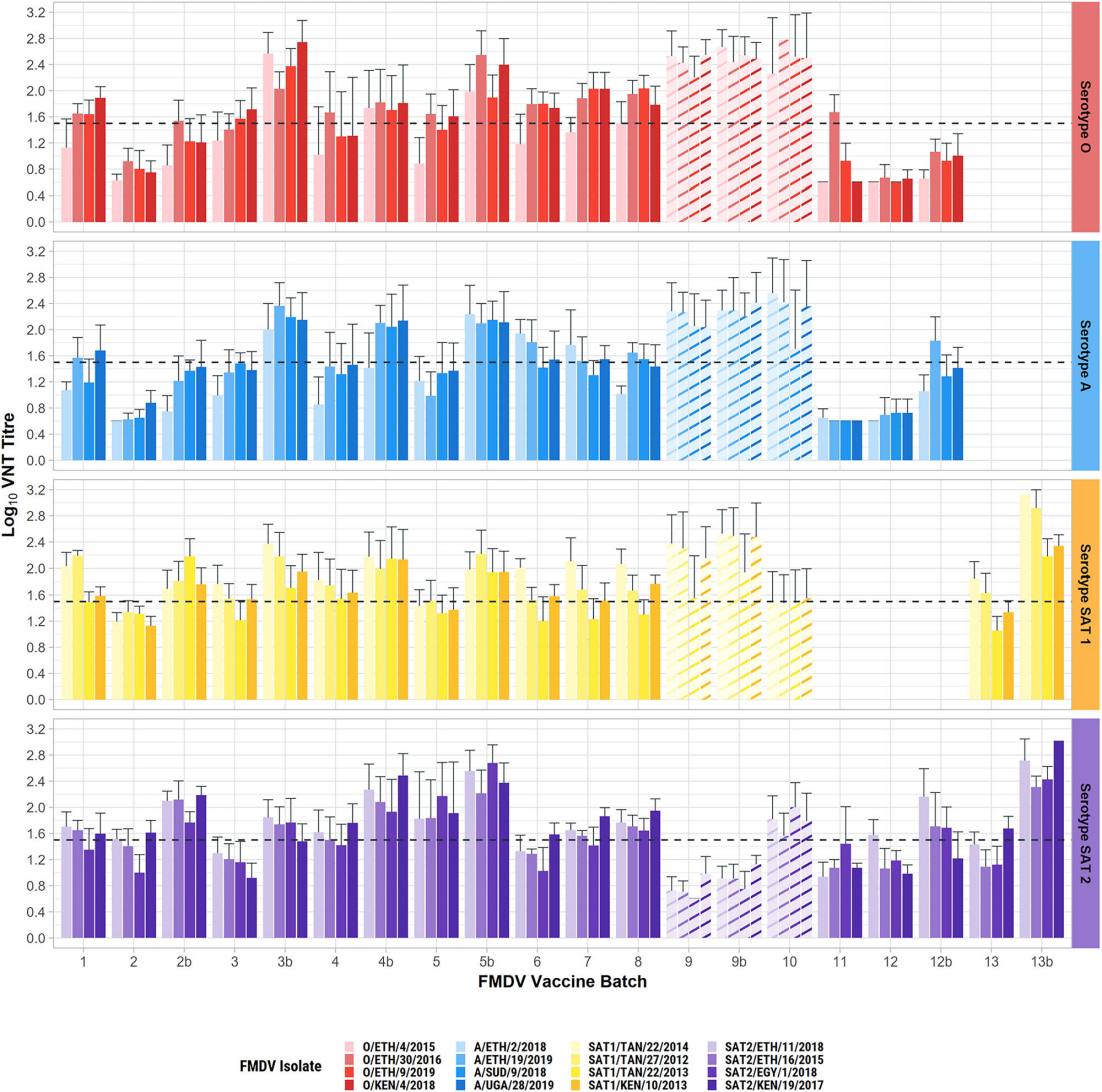
VNT titres elicited by 13 vaccine batches against the 16 reference panel viruses. Error bars show the standard deviation of mean values. Dotted line at log_10_ 1.5 represents the central threshold for expectancy of 75% cross-protection^4^. The lowest serum dilution was 0.9, and titres below this have been attributed the value 0.6. The “b” suffix to the batch number indicates where a booster vaccination was given. Batches 11 and 12 did not contain antigens of serotypes SAT 1, and Batch 13 did not contain O/A antigens. Batch 9–10 results are faintly coloured to indicate results of doubtful (batch 9) or rejected (batch 10) validity due to concerns over the pre-vaccination immunological status of cattle (see text).

The results for neutralisation of the 16 panel viruses by cattle sera from each trial have been deposited in Dryad (see Data Availability) and are illustrated in Fig. 5 (excluding redacted results for batches 3–5, as above). Table 2 summarises the ability of the vaccines to elicit a ≥ 1 in 32 titre of neutralising antibody to at least three out of four strains per serotype, by at least 60% of vaccinated cattle. The impact of different VNT thresholds on the cross-neutralising spectrum of activity of post-vaccination sera is also shown in Supplementary Fig. 1. The weaker immunogenicity of batches 2, 3, 5, 9, 11, and 12 for some serotypes is evident even at the least stringent (1 in 16) cutoff. Indeed, batches 2, 11, and 12 induced weak antibody responses to most strains and serotypes. Batch 9 induced uniquely weak responses to SAT 2 (*P* < 0.05 for all batches, except batch 3 where *P* = 0.07) but better responses to other serotypes (serotype O: *P* < 0.05 for all batches, except batch 10 (*P* = 0.99); serotype A: *P* < 0.01 for all batches, except batch 6 (*P* = 0.16) and batch 10 (*P* = 0.99); serotype SAT 1: *P* < 0.05 for all batches except batch 1 (*P* = 0.60) and batch 8 (*P* = 0.13)), although the pre-vaccination immunological status of the cattle has not been verified. Batches 3–5 induced mixed results according to serotype. Booster vaccinations significantly (*P* < 0.05) enhanced the antibody responses for all serotypes and batches, except for serotypes O, A, and SAT 2 for batch 9 (*P* = 0.23, *P* = 0.12, and *P* = 0.06, respectively). The effect of boosting ranged from <0.25 log_10_ (serotype O, batch 9) to 1.25 log_10_ (serotype SAT 2, batch 13) (Supplementary Fig. 2). Without a booster, only batch seven met the criteria for neutralisation by at least 60% of cattle for at least 3 strains of all four serotypes at a 1 in 32 cutoff. Batches 3–5, but not 2 and 12, fully met these criteria after cattle were given a booster vaccination, and other batches, such as 6 and 8, might have done so if boosters had been administered and tested. The apparently good results for serotypes O and A for batch 10 are invalidated by evidence that the cattle had already acquired immunity to these serotypes before the trial began.

**Table 2:**
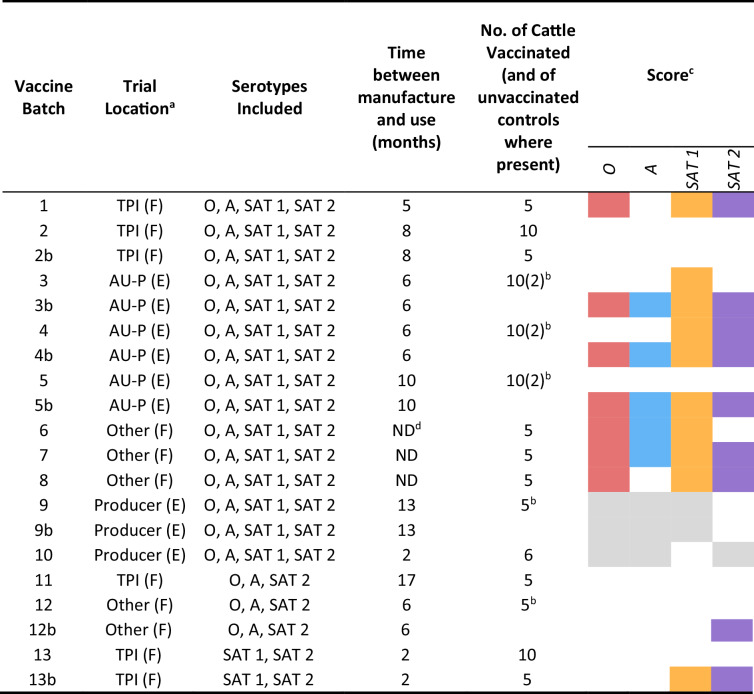
Study trials

^a^*TPI* The Pirbright Institute, *AU-P* AU-PANVAC, *(F)* FMD-free-country, *(E)* FMD-endemic country.

^b^Same cattle boosted.

^c^Coloured boxes show FMD vaccines that induced ≥1.5 log_10_ antibody responses in ≥60% of cattle for at least three reference strains for each serotype (grey colour indicates positive responses where day 0 results were not provided to confirm the validity of the trial).

^d^*ND* not disclosed.

Overall, some panel viruses were better and some more poorly neutralised by sera from vaccinated cattle, though which viruses were better or worse neutralised varied significantly amongst batches (*P* < 0.001 for all four serotypes). Combining the results from all trials and all panel virus tests, excluding the cattle with pre-vaccination immunity concerns (specific cattle from studies 3–5 and studies 9 and 10) the average percentage of cattle by batch that neutralised at or above the 1 in 32 (log_10_ 1.5) serological thresholds ranged from 29% (A/ETH/2/2018) to 84% (SAT 1/TAN/22/2014). The least neutralised panel viruses for each serotype (O/ETH/4/2015, A/ETH/2/2018, SAT 1/TAN/22/2013, SAT 2/ETH/16/2015) were also amongst the least well neutralised in the screening for the panel selection (Fig. 4). At the serotype level, with the same cutoff, the average percentages for neutralisation of serotypes O, A, SAT 1 and SAT 2 were 53%, 44%, 73%, and 66% respectively, which gives a ranking of vaccine immunogenicity by serotype as SAT 1 > SAT 2 > O > A. Some panel viruses of a given serotype showed similar reactivity patterns, suggesting that the number included could be reduced.

The VNT results of the guinea pig responses to vaccination are deposited in Dryad (see Data Availability), and the derived geometric mean titres are shown in Supplementary Table 2. Guinea pig serum titres were lower than those of cattle sera, and with the exception of neutralisation of two panel viruses in the responses of 1–3 guinea pigs to vaccine batch 5, only those receiving two doses of vaccine developed neutralising antibody responses above 1 in 16 (1.2 log_10_). An overall comparison of the geometric mean titres to each test virus was made for the 31-dpv guinea pig sera versus cattle receiving one or two doses of the same vaccine (Supplementary Fig. 3). This confirmed both a positive correlation and considerable variability.

## Discussion

A combination of genetic and antigenic screening was used to select a panel of 16 contemporary field isolates of FMDV, comprising four isolates per serotype, to be representative of the strains circulating in East Africa^12,13^. Using phylogenetics for the initial selection allowed many isolates to be screened and ensured that the predominant circulating lineages were represented. The final selection was verified by antigenic analyses since the analysed sequences only considered 1D/VP1 and not the entire capsid surface, and since many genetic differences are antigenically neutral, whilst a small number of specific amino acid substitutions can have a disproportionate effect on antigenicity^14^. Future methods of genetic selection could trim the regions aligned to focus on surface-exposed residues that are most likely to contribute to antigenically important epitopes.

Since testing by VNT is laborious, as small a panel as possible should be selected. This would be particularly the case for testing at vaccine batch level, where prolonged quality control procedures may delay the timely vaccine supply in emergency situations. As batch-to-batch variations are more likely to impact vaccine potency rather than antigenicity, a reduced panel may be appropriate here. Observation of the immunogenicity trial results shows that some isolates gave similar patterns of reactivity with sera against the vaccines evaluated. This suggests that a smaller panel of representatives per serotype could suffice, but would affect the acceptance criterion for the proportion of panel viruses required to be neutralised. It can be anticipated that the panel will need to be updated if new variants emerge or are introduced with different antigenic properties. This underlines the importance of continuing with routine surveillance for sampling and antigenic characterisation of FMD outbreak viruses.

If the virus panel is to be used in conjunction with serology to judge the adequacy of vaccine-induced immunity towards antigenically diverse viruses, then serological acceptance criteria are needed (even if not required for comparing the immunogenicity of vaccines). The correlation between in vivo and in vitro (i.e., serological) cross-protection has been analysed based on VNT results from post-vaccination cattle sera collected at the point-of-challenge from 18 in vivo studies involving heterologous virus challenge^8^. In two serotype O studies, high titres of antibody were required for cross-protection (log_10_ 2.3 mean value), but in the remaining studies, a mean VNT titre against the challenge strain of between 1.17 log_10_ (~1 in 16) and 1.67 log_10_ (~1 in 45) was associated with 75% protection^8^.

The results from the current immunogenicity studies were therefore examined using three cutoffs for the VNT, namely 1 in 32 (1.5 log_10_), bracketed by 1 in 16 (1.16 log_10_) and 1 in 45 (1.65 log_10_). Preliminary findings from the results presented by Gubbins et al. ^4^ had also informed the acceptance criteria chosen for 6 PD_50_ FMD vaccines for East African use, to be supported by the AgResults FMD Vaccine Challenge Project in Africa. In that project, vaccines are expected to induce neutralising antibody responses of at least 1 in 32 (1.5 log_10_) in at least three out of five cattle, for ≥70% (three out of four) of the panel viruses, after administration of one or two doses of vaccine, according to label requirements^11^. As vaccines may be supplied at different minimum potencies, usually to either ≥3 or ≥6 PD_50_^3^ (based on homologous challenge tests), more or less stringent acceptance criteria for expected cross-protection could be warranted. However, the serological cutoffs associated by Gubbins et al.^8^ with 75% protection roughly equate to a vaccine potency of 3 PD_50_, and from a practical viewpoint, this may be considered the minimum that is adequate and readily achievable for cross-protection. Even with the least stringent serological cutoff, not all of the vaccines are considered satisfactory, particularly since the study looked at peak immunity, which will wane by the time of revaccination.

FMD vaccines for immunogenicity studies were obtained from five manufacturers representing the principal suppliers to the East African market. As they were obtained under confidentiality agreements, the manufacturers cannot be identified. Purchasing vaccines sold on the open market could obviate this requirement, but carries the risk associated with difficulty assuring cold chain maintenance and associated vaccine integrity. One study was carried out by the manufacturer who supplied post-vaccination sera for testing (batches 9 and 10) from cattle in an FMD-endemic setting. For the other batches, testing was done by third parties in FMD-free settings (at Pirbright and two other European laboratories) or in an FMD-endemic setting (at AU-PANVAC). Compared to third-party immunogenicity trials, local trials carried out by the manufacturer do not require vaccine shipment, which may risk damage in transit. However, shipment is a necessity for normal vaccine supply, whilst the third-party trials have advantages of being independent, of following a consistent protocol with and without boosting, and of resulting in the collection and storage of large volumes of sera for future use in test development and calibration. When outbreaks due to the SAT 2 topotype XIV broke out recently in the Middle East^15^, following the likely introduction from East Africa, the availability of the sera from these trials enabled a rapid assessment of the suitability of the vaccines under study. This demonstrates that although the reference antigens are fixed, the overall approach facilitates a rapid response to new events.

Ensuring that only FMD naïve animals are used in immunogenicity studies can be a challenge in FMD-endemic countries. In this study, NSP screening failed to eliminate all cattle with pre-existing antibodies, and more stringent approaches to cattle selection procedures are required, such as only sourcing animals from farms with a reliable infection and vaccination history. If the first dose is administered in animals that have already been infected with FMDV or exposed to virus antigen, then booster-recall responses can be anticipated that will exaggerate the immunogenicity of the vaccine under test, as is likely to have occurred for the serotypes O and A, results of batch 10. Meanwhile, maternal antibodies may inhibit vaccine-specific responses, but their avoidance can be ensured by using cattle of at least six months of age. The use of a small animal model is a possible solution to the problem of obtaining truly seronegative cattle for immunogenicity studies of FMD vaccines in endemic settings. In a preliminary evaluation, we compared the neutralising antibody responses of guinea pigs vaccinated at the same time as cattle with batches 3–5. As expected^16^, there was a correlation between the responses, although in our study, only guinea pigs given two 0.2 ml doses responded to vaccination. More work is needed to optimise and standardise the guinea pig procedures (e.g., breed of guinea pig, dose and regime of vaccination) to confirm how closely and consistently their responses can predict protection in cattle.

Two principal questions arise when considering how to use heterologous panel VNT serology for evaluating FMD vaccines in practice. Firstly, what vaccine acceptance criteria are appropriate? Secondly, when and by whom should such testing be carried out? If the AgResults FMD Challenge criteria^11^ were adopted for the current findings, only one of the vaccines (batch 7) given without boosters elicited consistently adequate antibody responses for all serotypes and only three batches with a booster (batches 3b–5b) provided evidence of vaccine adequacy (Table 2, Supplementary Fig. 1). It is, however, likely that more of the examined vaccines would have met the standard if a booster dose had been used. Booster vaccination significantly enhanced antibody responses in these trials, with least effect for batch 9 where the second dose was given after a shorter interval of two weeks. As boosting is a permitted option under AgResults assessment, presumably, most manufacturers would opt to use it when obtaining evidence of their vaccine’s immunogenicity. However, giving a second dose extended the duration of the immunogenicity studies by 10 days and would not be an appropriate measure of efficacy where vaccines are applied in the field without a booster, as is common practice in East Africa. Furthermore, serological acceptance criteria have been mostly derived from live virus challenge of cattle after a single vaccination. After a two-dose vaccination regime, different immune mechanisms might alter this relationship.

If booster doses were avoided for the purposes of quality control, but the lowest serological cutoff was applied, then more of the vaccines evaluated could be considered acceptable. For example, with a threshold of 1 in 16 in VNT to be reached by at least 60% of singly vaccinated cattle, for at least 70% of panel viruses per serotype, then vaccine batches 4–8 would pass (Supplementary Fig. 1). To avoid specificity issues with such a low cutoff, it might be considered that only animals with pre-vaccination titres of less than 1 in 8 could be eligible, or else that a rise of at least twofold in VNT titre would be needed post-vaccination. The threshold for a vaccine to pass against ≥75% of strains for all serotypes might also be waived where there was sufficient evidence of the prevailing risks to consider that such broad coverage would not be needed. However, this would undermine the concept of broad-spectrum vaccines being suitable for the region and its complex epidemiology if a prophylactic vaccination is foreseen.

A potential issue in determining the proportion of cattle that would be expected to be protected (i.e., have a titre above the specified cutoff) is the small number of cattle used in the immunogenicity trials. Most of the batches included were tested in five cattle, so that the 95% confidence intervals for the proportion above the threshold are very wide and most include 60% protection (Supplementary Fig. 4). Increasing the number of cattle used in a trial would increase confidence in the estimate of the proportion protected. However, the width of the confidence interval is approximately proportional to 1/√*N*, where *N* is the number of cattle^17^. Consequently, to halve the width of the intervals would require four times as many cattle.

Another issue concerns the reliability of different potency assessments, including indirect measures derived from serology. Multiple factors contribute to variability that makes serological interpretation difficult^7^, such as: (i) the variability in antibody responses between different animals and in the amounts of antibody that different animals need for protection against different viruses; and (ii) the variability in the results when VNT is repeated, especially if testing is performed by different laboratories^8^. In this study, all the VN testing was performed in one laboratory with long experience of using the test under IEC/ISO17025 accreditation, but for these reasons, immunogenicity trials with small numbers of animals must be interpreted cautiously, as they cannot fully discriminate between good and poor vaccines. Effectively, this means that if strict acceptance criteria are adopted to ensure that only good vaccines pass, then a proportion of rejected vaccines will also have been adequate. Alternatively, use of laxer acceptance criteria will help to ensure good vaccines are not rejected, but will mean more poor vaccines being passed. Although this is discouraging, the results of the current study reveal large differences between some vaccine batches that are unlikely to be caused by chance or trial-specific factors, and many of which are obvious at all three of the evaluated cutoff titers. This suggests that even with a cautious interpretation, the approach is likely to be helpful, in practice, in eliminating poor-quality vaccines. A comparative evaluation and/or more general guidance that avoids simple pass or fail thresholds is another way to mitigate this problem. An example of a guarded interpretation is provided by that used for vaccine matching (also based on serological testing of small numbers of samples), the main alternative for evaluating antigenic relevance (e.g., similar to ref. ^18^).

**Fig. 6:**
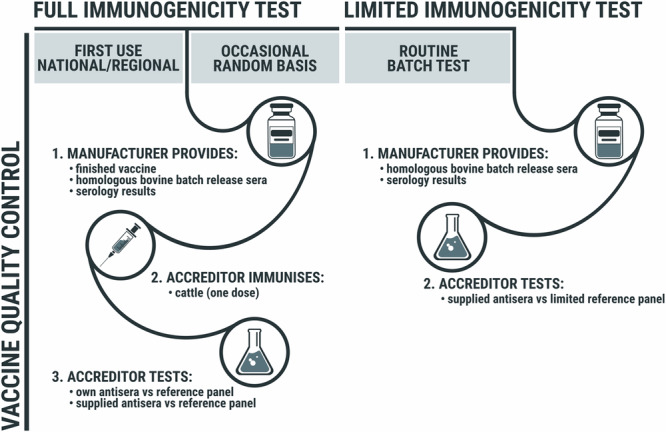
Possible pipeline for heterologous serology use in FMD vaccine QC. Circumstances where more or less rigorous approaches (full or limited) could be used for serological testing of vaccine potency using all or part of a reference antigen panel.

There are no vaccine banks in East Africa, and although a shift to prophylactic vaccination may be desirable, for the present, much vaccination is done reactively rather than as part of long-term preventive strategies. Therefore, vaccine requests are usually at short notice and associated with rapid delivery requirements. This reduces the time available for quality control of new vaccine batches and argues against time-consuming evaluations such as immunogenicity trials in animals followed by laborious serological testing of the derived sera, as might be the case for VNT with multiple panel viruses. While homologous vaccine batch tests of potency by immunogenicity should continue to be conducted by manufacturers, assessment of sera against a full panel of viruses by VNT is best reserved for registration of the product or pharmacovigilance, whilst other periodic third-party serological evaluation of batches can use sera derived mainly from manufacturer studies and using a subset of the full panel or even an ELISA for antibodies to FMDV structural proteins (SP-ELISA) to measure relative potency and not antigenic relevance (Fig. 6). Further work is needed to test the sera obtained in these trials by SP-ELISAs and to compare the results with those of VNT^19^, as well as to better harmonise VNT results from different laboratories. Batch release sera from immunogenicity trials conducted by vaccine producers are also invaluable to vaccine users in helping them to interpret studies of population immunity after vaccination^5^. However, pre-vaccination sera should also be supplied if the cattle came from an endemic setting, to verify that test animals were immunologically naïve at the outset. Use of such sera to test for neutralisation of viruses from reference panels can help to evaluate how well vaccinated populations are likely to be protected.

## Methods

### Selection of the East African FMDV reference Panel

#### Sequence comparisons

Five serotypes (O, A, SAT 1, SAT 2, SAT 3) and multiple strains circulate in East Africa^20,21^. However, SAT 3 is uncommon in domestic livestock and is not considered a priority for vaccination^21,22^. For this study, a total of 1180 VP1 sequences (O = 709; A = 138; SAT 1 = 143; SAT 2 = 190) were retrieved from the repository of FMDV sequences at the FAO FMD World Reference Laboratory (WRLFMD; www.fmdbase.org), the WOAH/FAO FMD Reference Laboratory Network, and GenBank. These represented lineages of the four serotypes of virus circulating in the region (Burundi, Democratic Republic of Congo, Eritrea, Ethiopia, Kenya, Rwanda, Somalia, South Sudan, Tanzania, and Uganda) along with genetically similar isolates from 16 other African countries and four Middle Eastern countries. Most of the viruses had been isolated between 2011 and 2019, but the SAT 1 viruses included isolates from 1999. For each serotype, the VP1/1D nucleotide and amino acid sequences were aligned in MAFFT 7.526^23^, and phylogenetic reconstructions were generated by maximum-likelihood (ML) method using IQ-TREE 2.3.6^24^. From the resulting nucleotide reconstructions, viruses were selected from the clades with contemporary representatives and grown in IB-RS-2 cell cultures. This yielded 63 genetically representative viruses with satisfactory growth characteristics (O = 22; A = 18; SAT 1 = 10; SAT 2 = 13). An unbiased greedy-approach was used to select four candidate reference viruses per serotype maximising the minimum phylogenetic distance between any pair of sequences included in the original ML trees^25^.

#### Verification of panel representativeness by antigenic typing

To check the antigenic distinctiveness and representativeness of the panel compared to more of the originally genetically selected viruses, all 63 isolates were compared by typing with murine monoclonal antibodies (Mabs). The Mabs used for antigenic typing had been prepared over many years at the WOAH FMD Reference Laboratory in Brescia, Italy, and included both neutralising and non-neutralising Mabs (Details deposited in Dryad (see Data Availability)). The assay used to analyse the reactivity of each virus with the serotype-homologous Mabs was a trapping ELISA, performed as previously described^26,27^, but using recombinant integrin αvβ6 as universal capture ligand for FMDV in place of serotype-homologous polyclonal rabbit immune sera^28,29^. Briefly, cell culture supernatants from the 63-passaged viruses were trapped using recombinant integrin αvβ6 coated onto the plates and then analysed against hybridoma supernatants for each serotype-homologous Mab; rabbit anti-mouse immunoglobulins conjugated to peroxidase were used as secondary antibody, and the reaction was developed with TMB (Surmodics) as substrate/chromogen. The reactivity was expressed as a percentage of the ELISA OD signal compared to a corresponding reaction with the parental FMDV strain. Optimal dilutions of viruses and Mabs, corresponding to saturating concentrations, were pre-determined by preliminary titrations.

For the neutralisation testing, in addition to the four candidate viruses that had been selected per serotype, a further three to five viruses per serotype were chosen using the same unbiased greedy approach cited above^25^. These 32 viruses (O = 7; A = 9; SAT 1 = 8; SAT 2 = 8) were tested for neutralisation by 198 archived bovine FMDV sera (48 Serotype O, 70 Serotype A, 14 SAT 1, 31 SAT 2, and 35 multivalent sera shared between SAT 1 and SAT 2). The sera had been collected from cattle immunised with 40 vaccines comprising different vaccine strains and strain combinations (Details deposited in Dryad (see Data Availability)). The VNT used was as described^6^.

Divisive hierarchical clustering was performed in R 4.2.3^30^ using the *cluster* package^31^ for computational analysis of the antigenic variability among and between the putative reference panel viruses using the antigenic data generated from both Mab typing and VNT procedures. The resulting clustering features for each FMDV serotype were plotted as heatmaps using the *ComplexHeatmap* package for R^32^.

#### Genome sequencing of the 16 reference viruses

Complete genome sequences of all 16 panel viruses were determined using an Illumina MiSeq platform as previously described^33^. Briefly, viral RNA was extracted from FMDV isolates using the RNeasy Mini Kit (QIAGEN Ltd., UK) and reverse transcription was performed using the Superscript III First-Strand Synthesis System (Thermo Fisher Scientific Inc., UK), both according to the manufacturer’s protocol. The resulting dsDNA samples were then used to prepare sequencing libraries with the Nextera XT DNA Sample Preparation Kit (Illumina Inc., UK), according to the manufacturer’s instructions. Reference assembly of the obtained raw paired-end reads to consensus-level sequences was performed using SeqMan Ngen and SeqMan Pro applications of Lasergene 16 (DNASTAR Inc., Madison, WI). The resulting consensus sequences were submitted to GenBank and received the accession numbers MT602077 to MT602092 (Table 1).

### Immunogenicity trials on vaccines for use in East Africa

#### Vaccines evaluated

Five manufacturers who sell FMD vaccines in East Africa agreed to provide a representative sample of their commercial vaccine stocks and were informed of the immunogenicity testing that would be undertaken. To facilitate participation, it was agreed that the trials would be reported in a similar way to laboratory proficiency tests, where a comparison of all results is made public, but the results of each participant remain anonymous except to themselves. The preference was for the supply of quadrivalent vaccine comprising antigens of serotypes O, A, SAT 1, and SAT 2. Four manufacturers supplied 11 vaccine batches (1–8 and 11–13) and one manufacturer provided bovine sera against two further batches (9 and 10). Each vaccine batch contained antigens of two or more of the above-mentioned four serotypes (Table 2—the suffix “b” indicates where a two-dose course of vaccination was given). Some vaccine batches included more than four strains of virus, and some included additional serotypes for which immunogenicity was not tested. Different batches from the same producer sometimes contained different strains. Two manufacturers used oil in the formulation of their vaccines (5 batches,) and the remainder supplied aqueous vaccine formulations. All vaccines were used within their shelf life, but at variable times (2–17 months) after manufacture.

#### Cattle trials

The animal studies funded by the project (trials of vaccine batches 1–5 and 11–13) were carried out independently of the vaccine producers and approved by the Ethical Review Committee of the Pirbright Institute as well as by local ethical committees for the studies not done at Pirbright (for batches 3–5 and 12). The procedures involving immunisation with commercially licensed products and periodic blood sampling were considered to be mild and not requiring anaesthesia. At the end of the experiments, animals were either released for reuse (AU-PANVAC) or killed by stunning and exsanguination (in FMD-free areas), which facilitated bulk blood collection. Although it is easier to source non-immune cattle in FMD-free areas, it is expensive to undertake the trials there, and it was desirable to study the feasibility of establishing them in local endemic settings. Randomly allocated unvaccinated cattle were only included for the studies conducted in Ethiopia (at AU-PANVAC, batches 3–5, where FMD has not been eradicated), to control for the risk of subclinical infection. Sera from vaccinated cattle were also supplied by two vaccine manufacturers (for batches 6–10), having been prepared as part of their quality control procedures and in compliance with oversight of their national regulatory authority. The numbers of cattle for each vaccine batch and trial (97 in all) and whether or not undertaken in an FMD-endemic or FMD-free-country are summarised in Table 2.

A minimum of five cattle is recommended for immunogenicity studies of FMD vaccines^11^. In each trial, groups of 5–10 cattle were vaccinated with a specific batch of FMD vaccine produced between 2019 and 2022. The vaccinated cattle were of various breeds, ages, and genders. All cattle were at least six months old and were monitored daily for general health. NSP ELISA (PrioCheck TM FMDV NS Ab^34^); was used to screen animals in endemic settings for undisclosed prior infection. At AU-PANVAC, the cattle were sourced at ~6 months of age from unvaccinated stock obtained from premises where FMD had not occurred in the previous 3 months. Shortly after arrival at the holding where the trial was conducted, the cattle were tested to determine their FMDV NSP status. Seronegative animals were separated. At least three weeks later, the cattle were retested to determine that all remained NSP seronegative, as evidence that FMDV had not been circulating in the group. Two of the 12 cattle selected for each study remained unvaccinated to control for intra-study seroconversion due to infection. None of the other trials included unvaccinated control cattle. For the cattle vaccinated by the manufacturer, NSP serology was also used to exclude animals with undisclosed infection, and antibodies were checked by NSP ELISA at purchase, on the day of vaccination, and at the end of each study.

To ensure the cold chain was maintained, temperature loggers were used for international vaccine shipments. Vaccination in the neck by the subcutaneous (four suppliers) or intramuscular (one supplier) route with 2–3 ml of vaccine, followed the manufacturers’ recommendations. Cattle received one or two doses of vaccine, with blood samples collected at 21 days after the first vaccination (dpv). Where given (seven batches), the second dose (booster) vaccination was usually at 21 days post-vaccination (dpv followed by blood sampling at 10 days after revaccination (dprv). However, for batch 9, cattle were revaccinated at 14 days after the first vaccination, with a final blood collection 21 days after the first vaccination. Sera were available for testing from blood collected at the time of vaccination (i.e., pre-vaccination sera) for nine batches of tested vaccines, four batches being given to cattle kept in FMD-endemic countries and five to cattle in FMD-free countries. Five pre-vaccination sera collected at The Pirbright Institute, UK, from a similar study of an additional monovalent vaccine batch were included in analyses of VNT specificity. Serum samples can be made available on request, subject to availability, given their finite volumes.

#### Comparative study on immunisation of guinea pigs

As a preliminary trial, the three vaccine batches tested at AU-PANVAC in cattle (batches 3–5, Table 2) were also given at the same time to guinea pigs at a dose of 0.2 ml administered intramuscularly. The studies were approved by the animal ethics committees of The Pirbright Institute and AU-PANVAC and employed 64 guinea pigs, of which 18–20 were vaccinated per batch. Half received a single dose and were killed by stunning and exsanguination 21 days later, and half were revaccinated after 21 days and killed by stunning and exsanguination 10 days later (31 dpv). At least one randomly selected guinea pig remained unvaccinated per vaccine batch tested (actually 1 for batch 3 kept till the boosted guinea pigs were killed; 4 for batch 4, of which 2 were killed at each (unboosted and boosted) end-point; 2 for batch 5 kept till the boosted guinea pigs were killed). The sera were tested by NSP serology and for their in vitro neutralisation of the panel viruses, as per the cattle sera, except that for vaccine batch 3, where, for economy, only one of the panel viruses was tested per serotype.

### Virus neutralisation testing

Immunogenicity trial sera were stored at −20 °C and tested at WRLFMD by VNT against the viruses in the East African Virus Panel. Sera were titrated by doubling dilutions from 0.9 log_10_ to 3.0 log_10_, and the test method followed the description in the WOAH Manual^9^. The Spearman Kärber method was used for titre calculation. Titre values of <0.9 log_10_ and ≥3.0 log_10_ were assigned values of 0.6 and 3.2 for analysis. VNT results for sera collected on the day of vaccination (day 0 or pre-vaccination sera) were compared for trials conducted in FMD-free versus FMD-endemic settings to determine the relative specificity of the testing in these settings; lower apparent specificity in endemic settings being an indicator of possible pre-existing immunity. Absence of a twofold or greater rise in titre (seroconversion) between day 0 and day 21 was considered indicative that pre-vaccination titres were non-specific, rather than due to prior infection or vaccination. Post-vaccination results were compared after use of three different cut-offs to score the degree of neutralisation of a given virus as adequate [≥1 in 16 (1.2 log_10_), ≥1 in 32 (1.5 log_10_), >1 in 45 (1.65 log_10_)] based on the range of titres associated with protection in cross-protection studies^4^. Selection of the central titre value (≥1 in 32 (1.5 log_10_) when demonstrated for at least 75% (three out of four) of strains in at least 60% (three out of five) of vaccinated cattle (Table 2) is a pragmatic approach allowing for the variability between outbred animals in antibody responses and protective thresholds^7,8,11^.

### Statistical analysis

Virus neutralisation titres at 21 dpv were analysed separately for each serotype using a linear mixed model. The response variable was log_10_ VNT, and the explanatory variables were vaccine batch and virus in the panel used to measure VNT as fixed effects and animal as a random effect. Model simplification proceeded via stepwise deletion of non-significant factors (*P* > 0.05) as determined by a likelihood ratio test, starting from a model including both explanatory factors and an interaction between them. The effect of boosting was also analysed separately for each serotype using a linear mixed model. The response variable was the change in log10 VNT between 21 dpv and 31 dpv, while the explanatory variables were vaccine batch as a fixed effect and animal as a random effect. The impact of the virus used in the panel was explored by incorporating it as a random effect, but its effect was not significant (models excluding it had an Akaike information criterion lower than that for models including it), so it was not included in the final analysis. In all models (day 21 titres and effect of boost), differences amongst batches were explored using Tukey post hoc tests, which correct for multiple testing. The assumption of normality was checked for the final models in each analysis by visual inspection of normal quantile plots and observed and fitted values plotted against the residuals. All analyses were implemented using the lme4^35^ and multcomp^36^ packages in R version 4.2.3^30^.

### Research animals

All animal studies were in accordance with relevant guidelines and regulations, and the cattle and guinea pig immunisations undertaken for this study were approved by The Pirbright Institute’s Ethical Review Committee in compliance with the Animals (Scientific Procedures) Act 1986, and licensed by The UK Home Office (https://www.pirbright.ac.uk/our-animal-research). Two commercial vaccine companies provided post-vaccination bovine sera collected as part of quality control procedures for vaccine batch release in accordance with the World Animal Health Organisation’s Standards (Chapter 3.1.8., https://www.woah.org/en/what-we-do/standards/codes-and-manuals/terrestrial-manual-online-access/).

## Supplementary information

SUPPLEMENTARY MATERIAL

## Data Availability

The genome consensus sequences were submitted to GenBank and received the accession numbers MT602077 to MT602092 (Table 1). All other data generated or analysed during this study, including the full VNT results, have been deposited in Dryad, along with descriptions of the monoclonal antibodies and antisera used for selecting the virus panel (https://datadryad.org/stash/share/xdzaW8RVLIMGyfLH3mHx8F9FrZXpHvfRJ1A-FL1IItk).
