# Supplementary material for: An antigen panel to assess the regional relevance of foot and mouth disease vaccines
Source: NPJ Vaccines. 2025 May 26;10:106. doi: 10.1038/s41541-025-01128-7 (PMC12106609; doi:10.1038/s41541-025-01128-7)

SUPPLEMENTARY MATERIAL

Supplementary Note 1.

Sero-status of cattle at vaccination

The cattle sourced in FMD endemic settings were all confirmed NSP ELISA negative to rule out prior undisclosed infection with FMDV. Nevertheless, the sera collected from these animals at AU-PANVAC on the day of vaccination were more reactive in VNT than their equivalents from FMD-free setting cattle (see table below) and on retest three were found to be NSP ELISA positive (51-64 percent inhibition; cut-off is 50%). As only one day 0 serum from an FMD-free location neutralised at 1 in 32 and none at higher titres, a precautionary rule was applied and results from cattle immunised at AU-PANVAC with one or more titres at or above 1 in 32 were excluded from subsequent analysis of vaccine immunogenicity. Considering the results of the reactor cattle in more detail, the evidence of their prior immunisation was mixed. Five batch 3 cattle were excluded due to day 0 sera with neutralising antibody titres of between 1 in 32 and 1 in 64, of which two scored positive on a NSP ELISA retest. However, these cattle did not show a day 21 booster response to the strains neutralised at day 0. One batch 4 animal was excluded for a 1 in 45 titre on day 0 and five batch 5 cattle were excluded, the strongest reactor having a day 0 SAT 2 titre of 1 in 128 and a positive NSP ELISA retest result.

Pre-vaccination sera were not available to test from studies 6-9, of which study 9 was carried out in an FMD endemic country, where the possibility of pre-existing immunity cannot be discounted. Batch 10, given to cattle by the same manufacturer as batch 9 in an endemic setting, gave rise to pre-vaccination sera that were tested 96 times against the panel viruses. The numbers of positive results generated at the three cut-offs were even higher than at AU-PANVAC (Supplementary Table 1) and all of the cattle had pre-vaccination titres ≥1 in 32 in at least one test. At 1 in 45, eight tests on pre-vaccination sera were positive for serotype O and 10 for serotype A, whereas only 1 positive result was obtained for the other two serotypes. Five and seven of the test results for serotypes O and A were positive at ≥1 in 64 respectively. Considering the NSP ELISA negative screening results, it is considered likely that the cattle had been previously vaccinated for serotypes O and A and the results cannot be considered as a valid basis for assessing vaccine immunogenicity; also casting doubt on the validity of the trial of batch 9.

Supplementary Table 1. VNT results on pre-vaccination sera.

Facility and location where cattle vaccinated	No. pre-vaccination sera	Total VNT performed	No. positive (% apparent specificity) at different cut-offs		
			$\geq$1 in 16	*$\geq$1 in 32*	*$\geq$1 in 45*
Independent facilities in FMD-free countries	40	460	18 (96.1)	1 (99.8)	0 (100)
AU-PANVAC, FMD-endemic country	36	576	81 (85.9)	22 (96.2)	11 (97.7)
Manufacturer, FMD-endemic country	6	96	41 (67)	25 (74)	19 (80)

Suplementary Table 2. Geometric mean titres (log_{10}) of cattle and guinea pigs that were vaccinated with common vaccine batches (after removal of cattle excluded due to non-negative prevaccination serology status).

Reference viruses tested by VNT

	Vaccine batch 3			
Sera	O KEN/4/2018	A UGA/28/2019	SAT 1 TAN/22/2014	SAT 2 KEN/19/2017
Cattle (21dpv)	1.80	1.40	1.85	0.89
Gpig (31dpv sera)	1.40	1.20	1.20	0.90
Cattle (31dpv)	1.75	2.13	2.37	1.46

	Vaccine batch 4															
Sera	O ETH/4/2015	O ETH/30/2016	O ETH/9/2019	O KEN/4/2018	A ETH/2/2018	A ETH/19/2019	A SUD/9/2018	A UGA/28/2019	SAT 1 TAN/22/2014	SAT 1 TAN/27/2012	SAT 1 TAN/22/2013	SAT 1 KEN/10/2013	SAT 2 ETH/11/2018	SAT 2 ETH/16/2015	SAT 2 EGY/1/2018	SAT 2 KEN/19/2017
Cattle (21dpv)	0.90	1.56	1.18	1.18	0.81	1.38	1.27	1.37	1.76	1.69	1.48	1.62	1.57	1.46	1.41	1.78
Gpig (31dpv sera)	0.89	1.20	1.17	1.43	0.60	1.79	1.58	1.37	1.56	1.15	1.15	1.43	1.20	0.92	1.22	1.17
Cattle (31dpv)	1.63	1.73	1.61	1.71	1.41	2.09	1.98	2.08	2.17	1.98	2.13	2.13	2.22	2.04	1.93	2.45

	Vaccine batch 5															
Sera	O ETH/4/2015	O ETH/30/2016	O ETH/9/2019	O KEN/4/2018	A ETH/2/2018	A ETH/19/2019	A SUD/9/2018	A UGA/28/2019	SAT 1 TAN/22/2014	SAT 1 TAN/27/2012	SAT 1 TAN/22/2013	SAT 1 KEN/10/2013	SAT 2 ETH/11/2018	SAT 2 ETH/16/2015	SAT 2 EGY/1/2018	SAT 2 KEN/19/2017
Cattle (21dpv)	0.65	1.49	1.14	1.40	1.00	0.81	1.16	1.37	1.32	1.34	1.12	1.13	1.40	1.49	1.79	1.47
Gpig (31dpv sera)	1.26	1.26	1.10	1.76	0.74	1.15	1.15	1.17	1.34	1.68	1.06	1.40	1.42	1.34	2.09	0.92
Cattle (31dpv)	1.88	2.44	1.81	2.30	2.13	2.12	2.09	2.13	2.03	2.25	1.90	1.95	2.45	1.95	2.76	2.19

Supplementary Figure 1. Cattle immunogenicity trial outcomes by serotype for each numbered vaccine batch (1-13 where suffix "b" indicates cattle received a booster vaccination). Each graph shows the number of reference strains (out of 4 per serotype) neutralised by at least 60% of vaccinated cattle at each of three possible VNT titre cut-offs (1 in 16, 1.2 log_{10}; 1 in 32, 1.5 log_{10}; 1 in 45, 1.65 log_{10}). Trivalent and bivalent vaccines only tested against constituent serotypes (batches 11-12: O, A, SAT 2; batch 13: SAT 1, SAT 2). Batch 9-10 results are faintly coloured to indicate results of doubtful (batch 9) or rejected (batch 10) validity due to concerns over pre-vaccination immunological status of cattle.

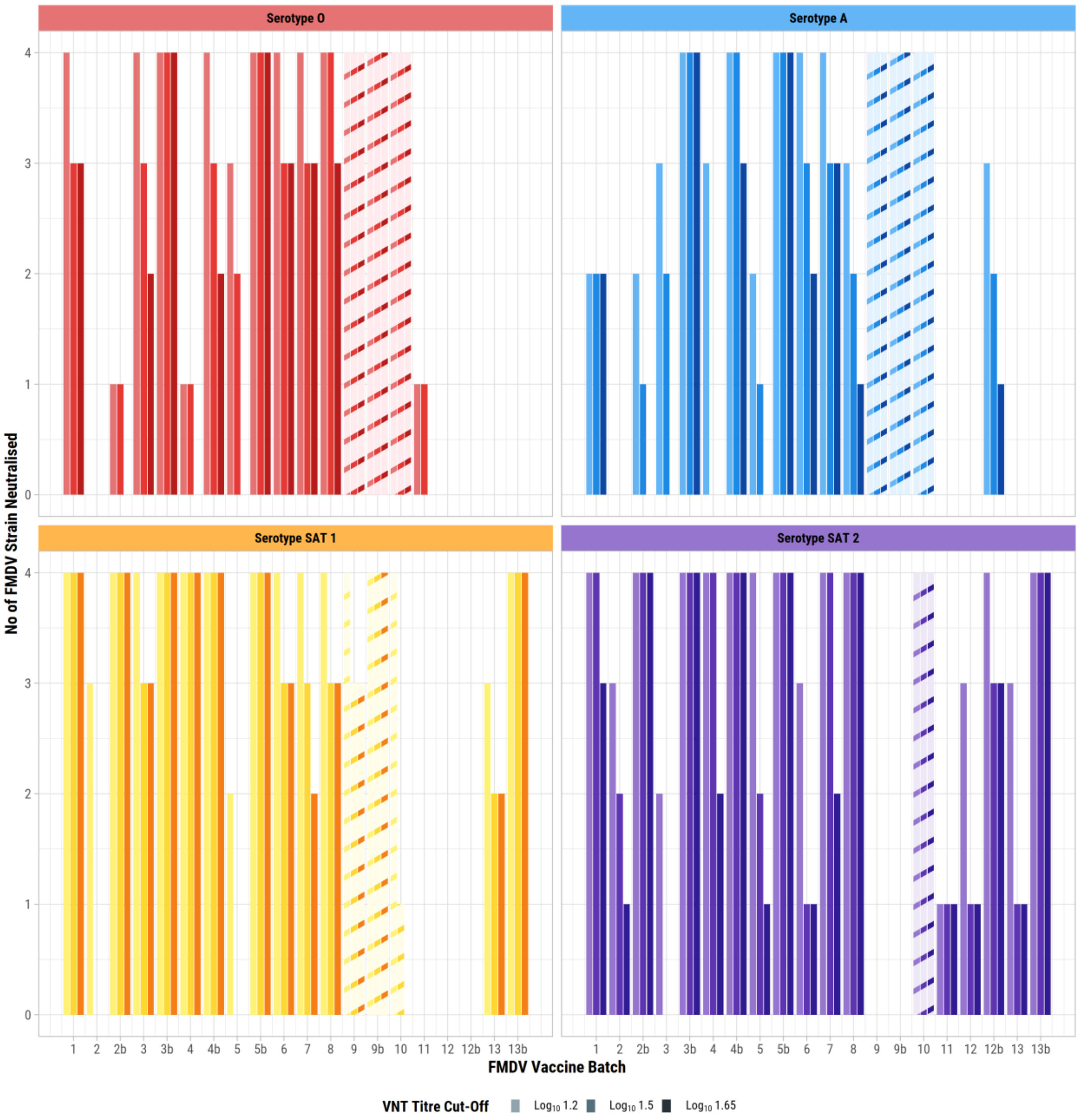

Supplementary Figure 2. Cattle booster effect. Change in $\log_{10}$ VNT associated with a booster vaccination. Plots show the estimated change (bars and circles) and the 95% confidence intervals (error bars).

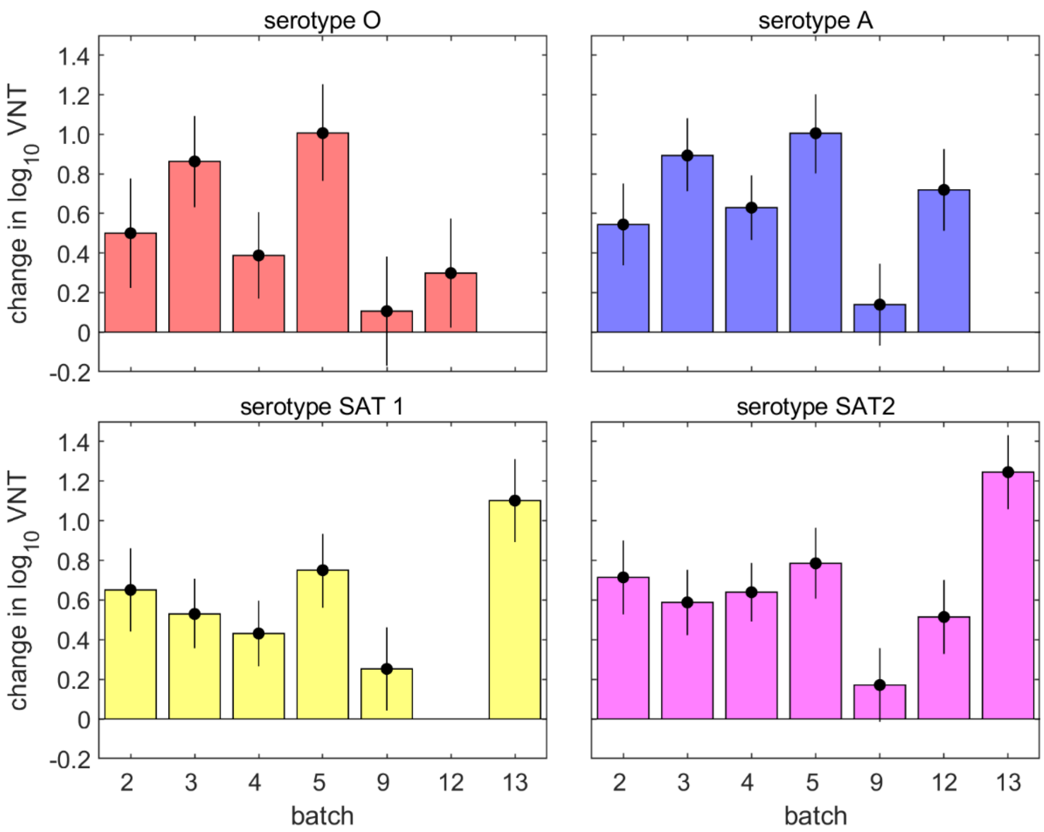

Supplementary Figure 3. Scatter plots of geometric mean titres for guinea pigs and cattle after immunisation with vaccine batches 3, 4 and 5. Log_{10} VNT titres of guinea pigs (y axis) ten days after second vaccination and cattle (x axis) 21 days after first vaccination (upper panels) or ten days after second vaccination (lower panels). Neutralisation is of the FMDV reference panel viruses (all sixteen, left hand panels) or by serotype.

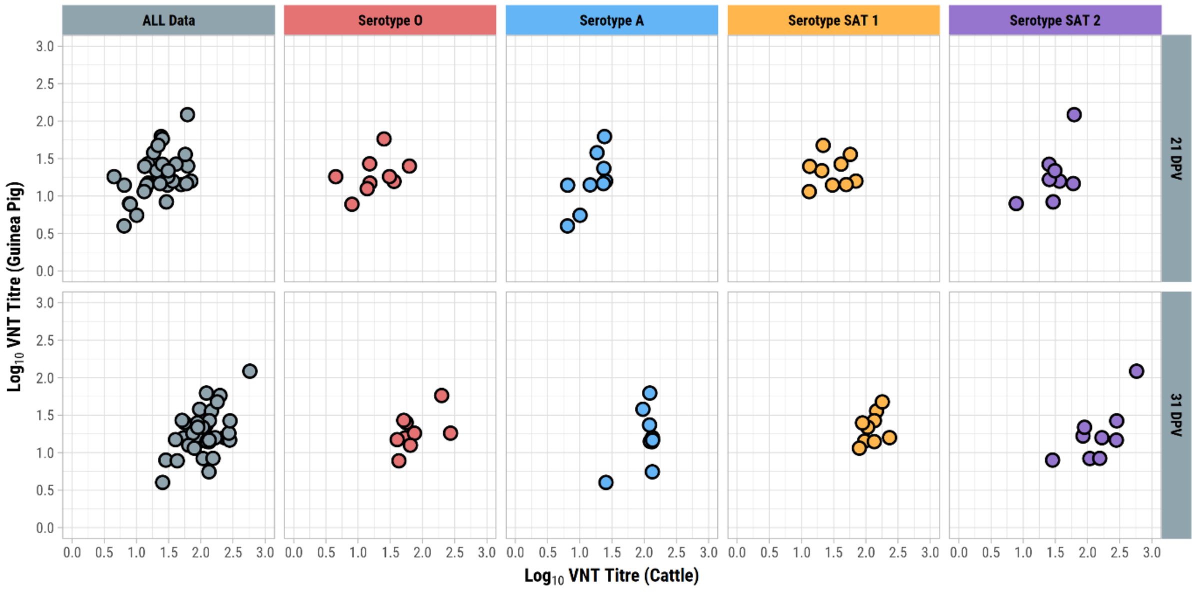

Supplementary Figure 4. Cattle immunogenicity trial outcomes by serotype for each numbered vaccine batch (1-13) at 21 days post vaccination. The plots shows the proportion of cattle that neutralised each reference strain (estimate (symbol) and 95% confidence interval) at each of three possible VNT titre cut-offs (1 in 16, 1.2 $\log_{10}$; 1 in 32, 1.5 $\log_{10}$; 1 in 45, 1.65 $\log_{10}$) for each serotype (O - red; A - blue; SAT 1 - yellow; SAT 2 magenta). Trivalent and bivalent vaccines were only tested against constituent serotypes (batches 11-12: O, A, SAT 2; batch 13: SAT 1, SAT 2). The dotted line shows the threshold at 60% protection.

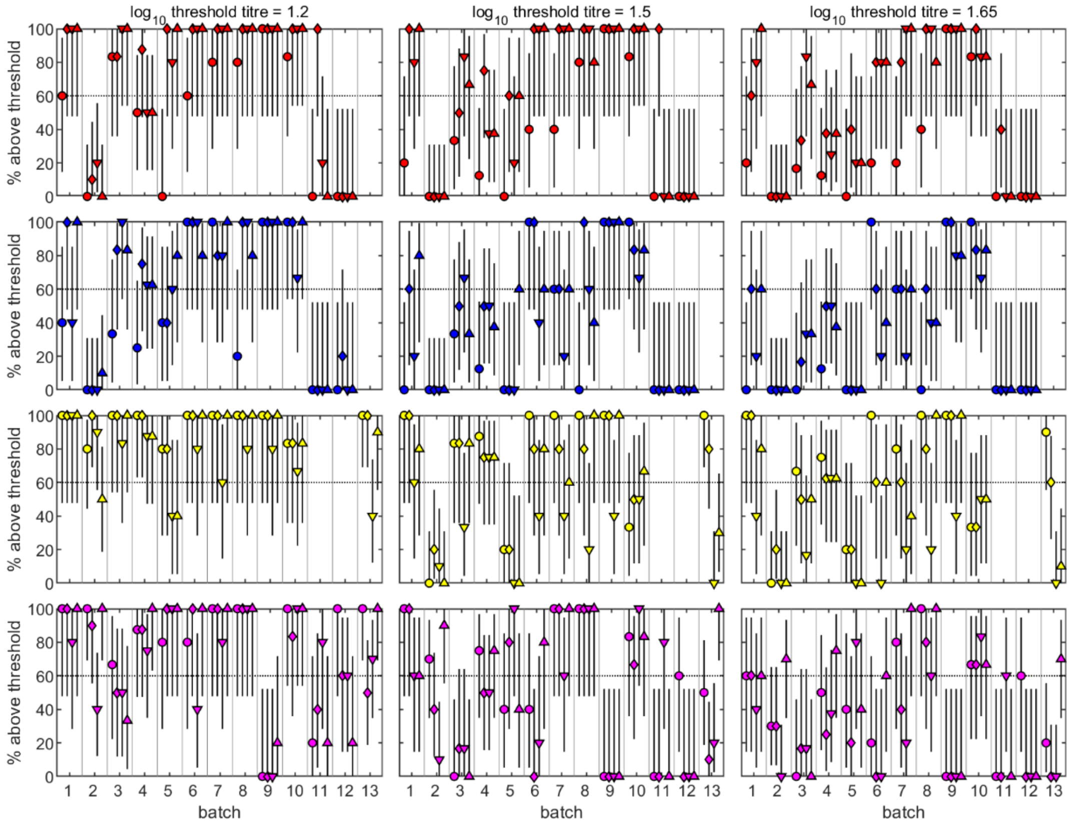

Supplement: Supplementary file 1 — SUPPLEMENTARY MATERIAL [file 41541_2025_1128_MOESM1_ESM.pdf]
